# Hyperconserved Elements in Human 5′UTRs Shape Essential Post-transcriptional Regulatory Networks

**DOI:** 10.3389/fmolb.2020.00220

**Published:** 2020-08-28

**Authors:** Paola Zuccotti, Daniele Peroni, Valentina Potrich, Alessandro Quattrone, Erik Dassi

**Affiliations:** Department of Cellular, Computational and Integrative Biology, University of Trento, Trento, Italy

**Keywords:** post-transcriptional regulation, 5cpsdummy′UTR, phylogenetic conservation, regulatory networks, RNA-binding proteins, homeobox, RBMX

## Abstract

Post-transcriptional regulation (PTR) of gene expression is a powerful determinant of cellular phenotypes. The 5′ and 3′ untranslated regions of the mRNA (UTRs) mediate this role through sequence and secondary structure elements bound by RNA-binding proteins (RBPs) and non-coding RNAs. While functional regions in the 3′UTRs have been extensively studied, the 5′UTRs are still relatively uncharacterized. To fill this gap, we used a computational approach exploiting phylogenetic conservation to identify hyperconserved elements in human 5′UTRs (5′HCEs). Our assumption was that 5′HCEs would represent evolutionarily stable and hence important PTR sites. We identified over 5000 5′HCEs occurring in 10% of human protein-coding genes. These sequence elements are rather short and mostly found in narrowly-spaced clusters. 5′HCEs-containing genes are enriched in essential cellular functions and include 20% of all homeotic genes. Homeotic genes are essential transcriptional regulators, driving body plan and neuromuscular development. However, the role of PTR in their expression is mostly unknown. By integrating computational and experimental approaches we identified RBMX as the initiator RBP of a post-transcriptional cascade regulating many homeotic genes. This work thus establishes 5′HCEs as mediators of essential post-transcriptional regulatory networks.

## Introduction

Post-transcriptional control of gene expression (PTR) is a key determinant of protein levels and of the consequent cell phenotypes (Vogel et al., 2010; Schwanhäusser et al., 2011). The 5′ and 3′ untranslated regions of the mRNA (UTR) mediate this role through sequence and secondary structure elements, bound by RNA-binding proteins (RBPs) and non-coding RNAs (ncRNAs). These factors ultimately control the fate of a transcript by regulating its cytoplasmic lifecycle (Glisovic et al., 2008; Bartel, 2018). RBPs are a major player in PTR, counting over 1500 human genes (Gerstberger et al., 2014) whose action forms a complex network of cooperative and competitive interactions (Dassi, 2017).

Several works have focused on characterizing regulatory elements in 3′UTRs, while less is known about functional regions in 5′UTRs. Which are the factors binding them, and which is their impact on the fate of the transcripts carrying them? A comprehensive catalog of functional regions in the 5′UTRs is still missing, hampering our ability to understand the mechanisms exploiting this regulatory hotspot. We and others have successfully compiled such a catalog in 3′UTR by exploiting the intuitive concept that evolutionarily conserved regions in mRNAs may be functional (Bejerano, 2004; Sathirapongsasuti et al., 2011; McCormack et al., 2012; Dassi et al., 2013). The 5′ and 3′UTR appear to have different functions (Hinnebusch et al., 2016; Mayr, 2017) and are thus likely endowed with distinct profiles of *cis*-elements and targeting *trans*-factors. However, this phylogenetic approach is general.

We present here the analysis of hyperconserved regions in 5′UTRs (5′HCEs), based on a broad set of 44 vertebrate genomes. Among the 5248 identified 5′HCEs are several known and highly conserved regulatory sites, indicating the sensitivity of our approach. We find homeotic genes enriched among 5′HCE-containing genes. They are key developmental regulators (McGinnis and Krumlauf, 1992; Philippidou and Dasen, 2013), highly conserved from fungi to mammals (Holland, 2013). Homeotic genes define embryonic regions identity (Mallo et al., 2010; Gehring, 2012) and affect organ, neural, and muscular development (Philippidou and Dasen, 2013; Zagozewski et al., 2014). Their transcriptional regulation is pretty well characterized (Mallo and Alonso, 2013), while little is known about 3′UTR regulation by RBPs (Pereira et al., 2013; Rogulja-Ortmann et al., 2014) or miRNAs (Yekta et al., 2004; Li et al., 2014; Wang et al., 2014). Limited evidence supports alternative transcription initiation sites or IRESes (Regadas et al., 2013; Xue et al., 2015) and regulation by RBPs through their 5′UTR (Nie et al., 2015). Among potential post-transcriptional regulators of homeotic genes, RBMX is an RBP associated with neuromuscular developmental defects in *X. laevis* (Dichmann et al., 2008) and *D. rerio* (Tsend-Ayush et al., 2005). RBMX regulates splicing (Heinrich et al., 2009; Wang et al., 2011) and transcript abundance (Liu et al., 2017).

Through this work, we describe a post-transcriptional regulatory network of homeotic genes shaped by 5′HCEs and driven by RBMX.

## Methods

### *5*′HCE Identification

All human 5′UTRs, the 44-vertebrates alignment (phastCons44way table) and sequence conservation scores (SCS) were obtained from UCSC (hg18 genome) (Tyner et al., 2017). Branch length score (BLS) was computed for each human 5′UTR as described in Dassi et al. (2013) and averaged with SCS to derive the hyperconservation score (HCS). Then, a sliding-window algorithm, starting with fully conserved 5-nucleotides seeds (HCS = 1.0) and expanding them upstream and downstream until a score threshold of 0.85 is reached, was applied to the 5′UTRs as described in Dassi et al. (2013), obtaining hyperconserved elements (HCEs). The threshold of 0.85 was chosen to have each score component (SCS and BLS) not lower than 0.7 when the other is 1.0 and vice versa, thus ensuring that HCEs are only the most conserved regions. Identified regions were then lifted over to the hg19 assembly and checked for consistency and being in a 5′UTR. Functional enrichments were computed by DAVID (Huang et al., 2007) using Gene Ontology, INTERPRO, PFAM, and SMART annotations. The full catalog of 5′HCEs has been added to the AURA2 database (Dassi et al., 2014), reachable at http://aura.science.unitn.it.

### Motif Analysis

*De novo* motif search was performed on homeotic genes 5′HCEs with DynaMIT (Dassi and Quattrone, 2016), using Weeder (Pavesi et al., 2004) for sequence motifs (length 6–12 nts with 1–4 mismatches resp.; motif in ≥ 25% of sequences), RNAforester (Höchsmann et al., 2003) for secondary structure motifs (multiple alignment, local search mode), and “co-occurrence” as motif integration strategy. The best integrated motif from DynaMIT was used, keeping only positions with ≥ 10 supporting sequences, thus trimming the motif. Trimmed parts represent the lowly-supported ends of individual motifs with respect to the core shared by multiple motifs.

The PWMs for 193 RBPs were obtained from CISBP-RNA (Ray et al., 2013). Pearson correlation between PWMs and the motif was computed by TFBSTools (Tan and Lenhard, 2016). The RBMX PWM was matched against homeotic 5′HCE sequences with Biopython (Cock et al., 2009). Only PWMs ≥ 4 nts long were used, retaining matches with score > 70%.

### Cell Culture

HEK293 cells were cultured in DMEM with 10% FBS, 100 U/ml penicillin-streptomycin and 0.01 mM l-glutamine (Gibco, United States), and maintained at 37°C in a 5% CO_2_ incubator.

### RBMX Knock-Down and Overexpression

We performed *RBMX* knock-down as described in Matsunaga et al. (2012). We used *RBMX* siRNA-1 (5′-UCAAGAGGAUAUAGCGAUATT-3′) and siRNA-2 (5′-CGGAUAUGGUGGAAGUCGAUU-3′) for knock-down, and negative control siRNA S5C-060 (Cosmo Bio, Japan). 1.5 × 10^6^ HEK293 cells were seeded into two 10 cm Petri dishes and transfected with Lipofectamine 2000, using a mixture of both siRNA at 25 nM.

Full-length *RBMX* was amplified by PCR using HeLa cells cDNA and the Fw: 5′ GAGGCGATCGCCGTTGAAGCAGATCGCCCAGGAA 3′ and Rv: 5′GCGACGCGTCTAGTATCTGCTTCTGCCTCCC 3′primers. The amplified fragment was digested with the *Sgf*I and *Mlu*I restriction enzymes and cloned into the pCMV6-AN-His-HA plasmid (PS100017, OriGene, United States), obtain the pCMV6-HIS-HA-RBMX vector. The construct was confirmed by sequencing. HEK293 cells were transfected as described above, with 2 μg of pCMV6-HIS-HA-RBMX or the mock empty vector as control. RNA extractions were performed 48 h post-transfection. All experiments were run in biological triplicate.

### RNA Immunoprecipitation

Ribonucleoprotein immunoprecipitation (RIP) was performed in biological triplicate with HEK293 cells transfected with pCMV6-HIS-HA-RBMX or the mock empty vector. Cell extracts were resuspended in NT2 buffer (50 mM Tris–HCl pH 7.4, 150 mM NaCl, 1 mM MgCl_2_, 0.05% NP-40 supplemented with fresh 200 U RNase Out, 20 mM EDTA and a protease inhibitor cocktail), chilled at 4°C. Anti-HA magnetic beads (Pierce, United States) were saturated in the NT2 buffer (adding 5% BSA for 1 h at 4°C), then added to lysates. The immunoprecipitation was performed overnight at 4°C in gentle rotation conditions, the immunoprecipitate washed four times with NT2 and resuspended in the same buffer. RNA extraction was performed from 10% of the volume of both the input and immunoprecipitate, using TRIzol (Invitrogen, United States). *FYN* was used as positive and *HNRNPM* as negative control (Heinrich et al., 2009).

### Polysomal Profiling and RNA Extraction

Polysomal profiling was performed according to the protocol in Dassi et al. (2013). Cells were treated with cycloheximide, lysed in 300 μl lysis buffer and centrifuged for 5 min at 13,000 g and 4°C. Lysates loaded on a 15–50% linear sucrose gradient were centrifuged at 4°C for 100 min at 180,000 g (SW41Ti rotor, Beckman Coulter, United States), and 1 mL fractions collected by continuous monitoring absorbance at 254 nm. Total RNA was obtained by pooling 20% of each fraction.

To extract RNA, fractions were treated with 0.1 mg/ml proteinase K (Euroclone, Italy) for 2 h at 37°C. After phenol-chloroform extraction and isopropanol precipitation, RNA was resuspended in 30 μl RNase-free water. RNA was assessed by an Agilent Bioanalyzer and quantified by a Qubit (Life Technologies, United States).

### Western Blots

Ten percent of each sucrose gradient fraction was pooled (knock-down/overexpression validation) or processed separately (RBMX polysomes distribution) to extract proteins using TCA/acetone precipitation. Proteins were resolved on 15% SDS-PAGE, transferred to nitrocellulose membranes and immunoblotted with RBMX (Abcam, United Kingdom), *HA* (Bethyl Laboratories, United States) and RPL26 antibodies (Abcam, United Kingdom). Blots were processed by an ECL Prime detection kit (Amersham Biosciences, United Kingdom).

### RNA-Seq

Libraries were prepared in biological triplicate with 500ng RNA of each sample according to the manufacturer’s protocol. For the RIP and the RBMX knock-down we used a TruSeq Targeted RNA Custom Panel Kit (Illumina, United States), sequenced with a 50-cycle MiSeq Reagent Kit v2 (Illumina, United States) on a MiSeq machine. For the overexpression, we used the TruSeq RNA Sample Prep Kit (Illumina, United States), sequenced on six lanes at 2 × 100 bp on a HiSeq 2500 machine.

Reads were preprocessed (quality < Q30, adapters stripped) with trimmomatic (Bolger et al., 2014), then aligned to the hg38 genome (GENCODE v27 annotation) with bowtie2 (Langmead and Salzberg, 2012) or STAR (Dobin et al., 2013). Gene read counts were normalized by library size. Targeted RNA-seq samples with < 10 mapped reads per gene were discarded. RIP fold enrichment was computed for each replicate as (RIP_RBMX-IP/INPUT_RBMX-IP)/(RIP_HA-IP/INPUT_HA-IP). This targeted assay does not fit the assumptions of differential expression (DE) determination methods (reads distribution, fraction of DE genes), so we used a Wilcoxon test. For conventional RNA-seq, DE was computed with DESeq2 (Love et al., 2014). Polysomal samples were compared as siRBMX/CTRL and the same was done for total samples. A gene was then defined polysomal (or total) DEG if it exhibited a significant change in one level not matched by a significant change in the other. Differential exon usage was computed by DEXseq (Anders et al., 2012) (adjusted *p* ≤ 0.05 for both analyses).

## Results

### *5*′HCEs Are Short and Clustered Phylogenetic Footprints

To extract phylogenetically conserved regions from the 5′UTR of human mRNAs, we applied to the 5′UTRs the pipeline we previously described for 3′UTRs (Dassi et al., 2013). Briefly, we computed a per-nucleotide hyperconservation score (HCS), ranging from 0 to 1. The HCS is the average of sequence conservation and the fraction of the phylogenetic tree covered by that UTR alignment (branch length score). A sliding-window approach was then used to find 5-nucleotides seeds with maximum conservation. Seeds were eventually extended upstream and downstream along the UTR until HCS remained above a threshold of 0.85, thus extracting only highly conserved regions (see section “Methods”). We term these regions 5′ hyperconserved elements (5′HCEs).

**FIGURE 1:**
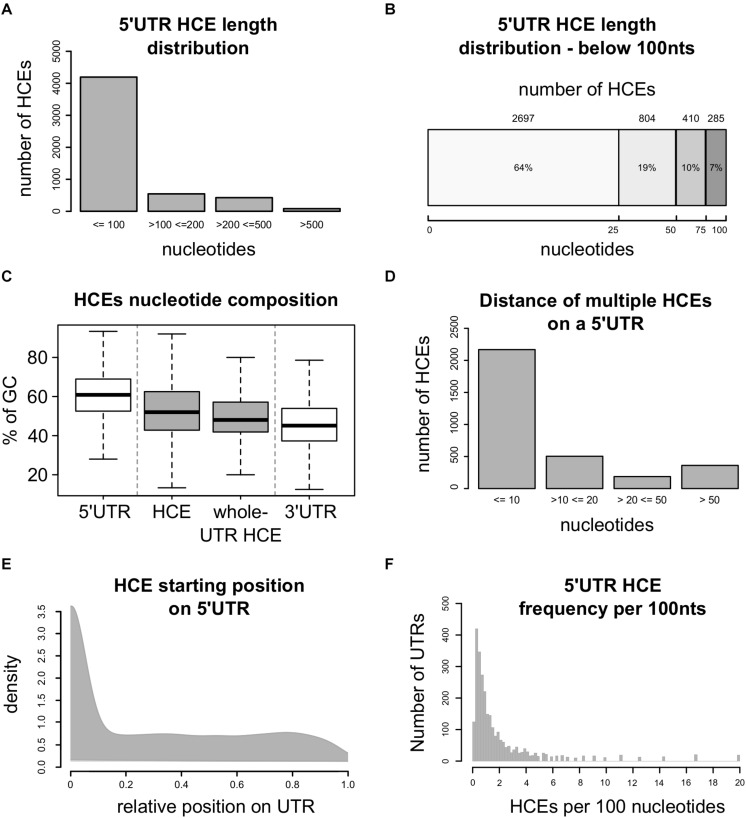
Homeotic genes are enriched in 5′HCEs. **(A)** Distribution of 5′ hyperconserved element (5′HCEs) lengths. **(B)** Detailed distribution of 5′ hyperconserved element (5′HCEs) lengths under 100 nucleotides. **(C)** Displays the density of AU and GC nucleotide frequencies in 5′HCEs with respect to whole 5′ and 3′UTRs, and to those 5′occupying their whole 5′UTR (*whole-UTR HCE*). **(D)** Shows the number of HCEs at a given nucleotide distance of one another in 5′UTRs carrying more than one of these elements. **(E)** density of relative HCE start positions on 5′UTRs (0 = UTR start, 1 = UTR end). **(F)** density of 5′HCEs per 100 nucleotides of HCE-containing 5′UTRs.

This approach led to the identification of 5248 5′HCEs (Supplementary Table 1), contained in the 5′UTRs of 2737 transcripts coding for 2228 genes. These HCEs are mostly short (Figure 1A), with an average length of 72 nucleotides and a median of 24 (Figure 1B). 5′HCEs are thus 28% shorter on average than 3′UTR HCEs (Dassi et al., 2013). However, one should consider that 5′UTRs are on average almost three times shorter than 3′UTRs (mean length of 455 nts for 5′UTRs and 1282 nts for 3′UTRs). We also computed length and abundance statistics for 5′HCEs at different HCS thresholds (every 0.05 from 0.85 to 0.5). The number of identified 5′HCEs (Supplementary Figure 1A) keeps decreasing from 0.5 to 0.85 (using 0.85 yields around half the 5′HCEs of 0.8), with a sharper decrease from 0.7 to 0.75. The mean length of 5′HCEs (Supplementary Figure 1B) is over 200 nts if using a threshold of 0.5 and progressively decreases up to a threshold of 0.7, where it flattens around the value found at 0.85 (slightly higher due to considerably smaller amount of 5′HCEs). When considering the distance between HCEs, it increases gradually from a mean of 21 nts (0.85) to 142 nts (0.5). This is due to close HCEs being merged (with a low score threshold few nucleotides are not enough to lower the HCE score below that threshold), and only HCEs separated by long stretches of lowly conserved nucleotides being kept as distinct HCEs. Globally, nucleotides in 5′HCEs represent only 1.47% of all 5′UTR nucleotides, making them rare regions. This value progressively increases by lowering the HCS threshold, reaching 13.7% at 0.7 HCS and 35.36% at 0.5 (Supplementary Figure 1C). We then analyzed the nucleotide composition of 5′HCEs. Figure 1C shows that 5′HCE are on average slightly less rich in GC than whole 5′UTRs (53% vs. 61%) and richer than 3′UTRs (46%), with no difference for 5′HCEs covering whole UTRs. To further characterize the properties of 5′HCEs, we also observed their relative positional distribution. As can be seen in Figure 1D, 40% of all 5′HCEs (2168/5248) are within 10 bases of another 5′HCE. This figure increases to 67% if we exclude the 2028 isolated 5′HCEs (i.e., only 5′HCE found on that given 5′UTR), and only a few (159, 3%) are more than 50 nucleotides away from another 5′HCE. When considering a maximum distance of 20 nucleotides between 5′HCEs, 2672 out of 3220 non-isolated 5′HCEs (82%) are found in clusters. This suggests the prevalence of a clustered 5′HCE organization, a pattern also observed for 3′UTR HCEs (Dassi et al., 2013). Eventually, we analyzed the position and density of 5′HCEs in the containing UTRs. We found them spread along the 5′UTR with a preference for its initial 10% (Figure 1E, 48% of HCEs). However, 27% of the 5′HCEs cover 95% or more of their 5′UTR and thus start around its first bases. When excluding these, this positional preference decreases to 18% of the 5′HCEs only. Eventually, HCEs are rather sparse in 5′UTRs, with a median density of 0.98 HCEs per 100 5′UTR nucleotides, and 75% of HCE-containing 5′UTRs having at most 2.3 such elements per 100 nucleotides (Figure 1F).

### *5*′HCE Genes Are Enriched in Essential Cellular Functions

**FIGURE 2:**
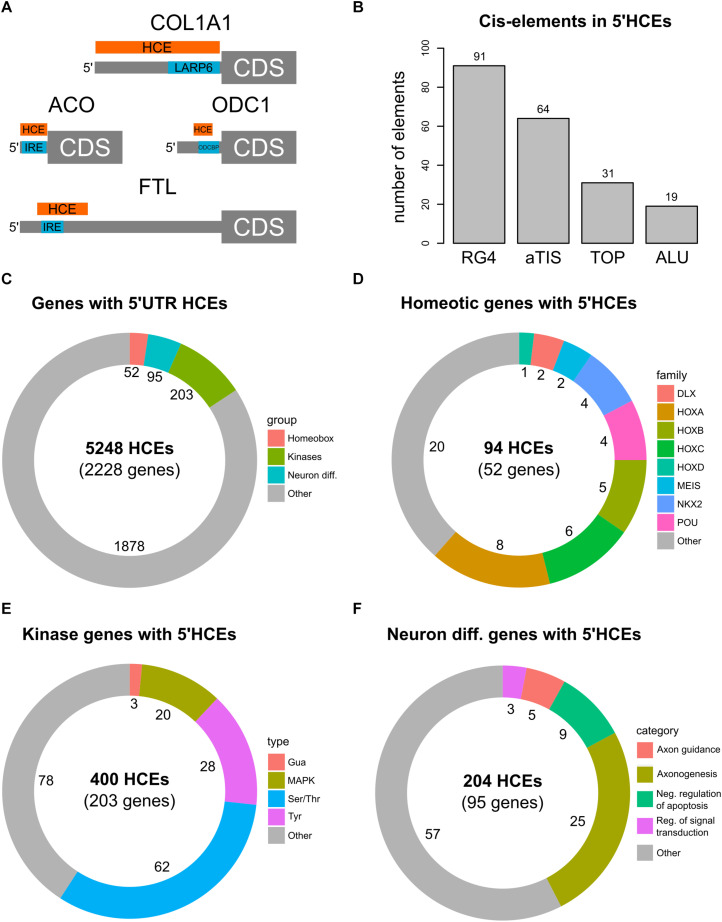
5′HCE genes are enriched in essential cellular functions. **(A)** instances of known conserved cis-elements and binding sites in 5′HCEs of the *ACO*, *COL1A1*, *FTL*, and *ODC1* 5′UTRs. **(B)** Overlap of 5′HCEs with 5′UTR cis-elements derived from the AURA2 database. **(C–F)** Distribution of 5′HCEs by functional gene groups for all 5′HCEs **(C)**, homeotic genes **(D)**, kinases **(E)** and neuronal differentiation genes **(F)**. The numbers next to each group show the related amount of genes.

We then investigated whether 5′HCEs represent functional regions in the 5′UTRs. We thus searched in the AURA2 database (Dassi et al., 2014) for 5′UTR cis-elements and RBP binding sites that were previously characterized and known to be highly conserved. In particular, as shown in Figure 2A, we first considered two iron response elements (IREs) (Hentze et al., 1987; Gray et al., 1996) in *FTL* and *ACO2* mRNAs. In both cases, an HCE in that 5′UTR contains the IRE. We then considered two conserved binding sites for the LARP6 and ODCBP RBPs, on *COL1A1* (Cai et al., 2010) and *ODC1* (Manzella and Blackshear, 1992) mRNAs. While for *COL1A1* a 5′HCE contains the LARP6 binding site completely, the overlap with *ODC1* is partial. Furthermore, 5′HCEs do not overlap with uORFs (Wethmar, 2014) or IRESs (Yamamoto et al., 2017).

We then overlapped 5′HCEs with the 10222 5′UTR-specific cis-elements in AURA2 (Figure 2B, including alternative translation initiation sites, ALUs, RG4s, and terminal oligopyrimidine tracts). Of these, only 205 (2%) overlap with 5′HCEs. We then generated 1000 sets of random 5′UTR portions with the same length distribution as 5′HCEs. Those contained at least as many elements as true 5′HCEs in 988 cases (*p* = 0.988), suggesting no enrichment of those specific *cis*-elements in 5′HCEs.

We then annotated genes containing one or more 5′HCEs by performing a functional enrichment analysis. The results revealed the presence of three highly enriched functional groups (Figure 2C and Supplementary Table 2). The first contains 52 homeobox genes (Figure 2D). These are highly conserved transcription factors, responsible for developmental patterns (Gehring, 2012; Philippidou and Dasen, 2013). The second group includes 203 protein kinases (Figure 2E). These represent various kinase types (Ser/Thr, Tyr, and others) affecting several signaling pathways (such as MAPK, NFKB, and others). A last, functionally broader group includes 95 genes implicated in neuronal differentiation and axonogenesis (Figure 2F).

Given the essentiality of homeotic genes in development and their high functional coherence, we focused our attention on this group. Among these 52 genes, containing 94 5′HCEs, are members of all four Hox clusters (Figure 2D, eight *HOXA*, five *HOXB*, six *HOXC*, and one *HOXD* genes). Other families are also included, such as *NKX* and *POU* (four genes each), *MEIS* and *DLX* (two genes each). All these proteins contain a homeobox domain and control developmental processes. Nevertheless, specific functions such as pattern specification (25 genes), cell motion (10 genes), and neuronal differentiation (20 genes) involve only a subset of genes. Homeobox 5′HCEs have a median length of 28 nucleotides (ranging from 5 to 423 nucleotides). They are often clustered, as 48/63 non-isolated HCEs are within 20 nucleotides of one another. The 52 genes and their functional annotation are listed in Supplementary Tables 3, 4.

### Homeotic *5*′HCEs Contain an RBMX-Binding Signature

**FIGURE 3:**
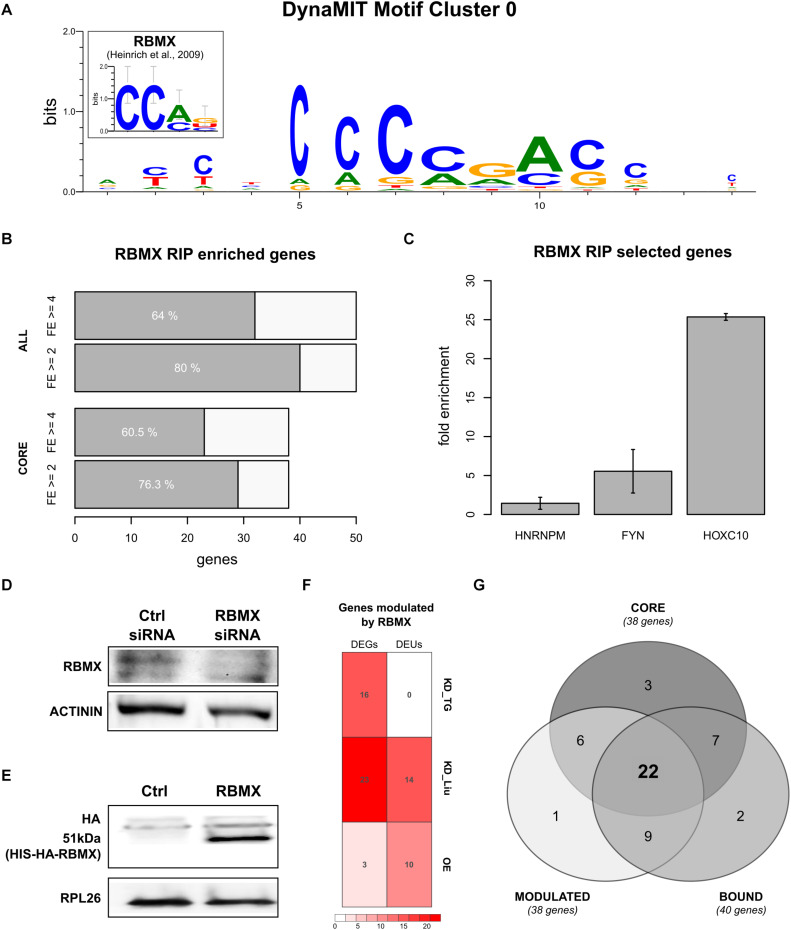
RBMX binds to homeotic genes mRNAs and post-transcriptionally controls their expression. **(A)** Best motif cluster identified by DynaMIT in homeotic 5′HCEs by integrating sequence and secondary structure motifs. The inset shows the known RBMX binding motif for comparison. **(B)** Number of genes enriched in the RBMX RIP assay, at fold enrichment threshold 2 (FE = 2) or 4 (FE = 4) in at least 2/3 replicates. Highlighted are the core HOX genes set (38 homeotic genes identified by our analysis as potentially bound by RBMX) and all tested genes (50 genes). **(C)** RIP fold enrichment for the negative control (*HNRNPM*), the positive control (*FYN*), and one representative of the HOX core genes set (*HOXC10*, gene with the lowest standard deviation among core genes having FE = 4 in all three replicates). **(D)** RBMX western blot in HEK293 cells treated with control and RBMX siRNA, with Actinin used as reference protein. Different parts of the gel are shown for the RBMX and control bands. Full blots are shown in Supplementary Figure 3A. **(E)** HA-tag western blot in control and RBMX-overexpressing HEK293 cells, with RPL26 used as reference protein. Different parts of the gel are shown for the RBMX and control bands. Full blots are shown in Supplementary Figure 3B. **(F)** Number of differentially expressed genes (DEGs) and differential exon usage (DEUs) in our RBMX knock-down targeted RNA-seq (KD_TG), the Liu RBMX knock-down dataset (KD_Liu) and our RBMX overexpression dataset (OE). **(G)** Intersections for the set of genes modulated by RBMX (MODULATED, defined as the union of DEGs and DEUs), the core set of homeotic genes (CORE), and genes bound by RBMX as per the RIP assay (BOUND).

We then studied whether homeotic genes could be controlled by a common regulatory mechanism through binding sites within their 5′HCEs. We performed an integrative motif search with DynaMIT (Dassi and Quattrone, 2016), combining a sequence search (Weeder; Pavesi et al., 2004) with an RNA secondary structure search (RNAforester; Höchsmann et al., 2003), and clustering motifs co-occurring on the same HCEs. The best resulting motif, shared by most homeotic 5′HCEs, is short, unstructured, and C-rich (Figure 3A). Breaking down the consensus by its composing motifs (Supplementary Figures 2A,B) reveals CGAC as a core for sequence search motifs and CCAG as secondary structure search consensus. The same search performed on all 5′HCE sequences yielded no significant secondary structure motif and three AG-rich sequence motifs (CGAAGA, GTCGAAGA, GGAGAAGAAG) which do not correspond to the homeotic 5′HCEs motif, suggesting the specific enrichment of the latter in homeotic genes.

We then tried to understand which trans-factor might be binding this motif to regulate homeotic genes expression. We thus performed a search on known RBP binding motifs using CISBP-RNA (Ray et al., 2013). The results highlighted a protein, RBMX, having a binding consensus strikingly similar to the homeotic 5′HCE motif (93.5% similarity and Pearson correlation 0.73, known motif shown in Figure 3A). We thus searched for the RBMX motif in homeotic 5′HCEs to systematically map potential binding sites. The results show potential RBMX binding sites spread along the homeotic genes HCEs within their 5′UTRs (Supplementary Figure 2C).

### RBMX Binds to Homeotic mRNAs

The association of RBMX with developmental defects (Tsend-Ayush et al., 2005; Dichmann et al., 2008) makes it a promising candidate post-transcriptional regulator of homeotic genes, as suggested by the motif we detected in the 5′HCEs of those mRNAs. So, we first assessed whether RBMX binds homeotic mRNAs. We performed an RNA immunoprecipitation (RIP) in HEK293 cells followed by targeted RNA-seq of the mRNAs of 50 genes. These included our 38 “core” homeotic genes (i.e., whose mRNA contains a 5′HCE and an RBMX-binding motif), further members of families in the core set, RBMX, and two controls. We computed a fold enrichment (FE) for each gene as the ratio between the normalized RIP abundance and the corresponding input abundance (Supplementary Table 5). We found 29/38 core genes (76%, Figure 3B) enriched at least twofold in at least two out of three replicates (23/38 with FE ≥ 4). Among all tested genes, 40 (80%) are enriched at least twofold in at least two replicates (32/50 with FE ≥ 4), including our positive control *FYN* (Figure 3C, average FE = 5.45), but not *HNRNPM* (negative control, average FE = 1.43) (Heinrich et al., 2009). Eventually, we compared our RIP results with targets found by a RBMX PAR-CLIP assay (Liu et al., 2017) and found 21/50 genes bound by RBMX, 17 of which are enriched in our RIP, thus validating those by a second experimental approach. Globally, this data shows that RBMX binds the mRNAs of many homeotic genes, thus likely contributing to their PTR. However, while 9 of these 17 genes contain a binding site in the 5′UTR according to the PAR-CLIP, we cannot determine conclusively that RBMX binds to the 5′UTR of all our genes, as the RIP assay cannot provide this information. Further work will thus be needed to confirm this aspect.

### RBMX Controls Homeotic mRNAs by Post-transcriptional Mechanisms

As RBMX binds to most homeotic mRNAs containing a 5′HCE, we studied whether this RBP can affect their expression. We used siRNAs to knock-down RBMX in HEK293 cells, reducing its protein level by 78% (Figure 3D, *t*-test *p* = 0.00214). Then, we performed a translatome profiling via sucrose gradient fractionation, followed by targeted RNA-seq of total and polysomal fractions for the 50 genes previously tested by RIP. We compared the silencing polysomal samples with the control polysomal samples and did the same for the total fraction samples. By doing so, we found 16 upregulated genes at the polysomal level (with no corresponding significant change at the total level) upon RBMX silencing (log2 fold change ≥ 1 and adjusted *p* ≤ 0.1). However, total replicates were variable (average Spearman correlation = 0.83), and this assay cannot detect alternative splicing, which RBMX may also modulate. So, we reanalyzed a HEK293 total RNA-seq assay after RBMX knock-down (Liu et al., 2017). Eventually, we complemented this dataset by overexpressing RBMX in HEK293 cells (Figure 3E) and performed a further translatome profiling followed by RNA-seq of total and polysomal fractions. We analyzed differentially expressed genes (DEGs) and differential exon usage (DEU) events (Figure 3F, adjusted *p* ≤ 0.05). DEGs were mostly in the knock-down (23/50 genes, 6 up- and 17 down-regulated at the total level in the Liu dataset), with only 3 genes in the overexpression dataset (2 up- and 1 down-regulated, 2 differentially expressed at both the total and polysomal level, 1 only at the polysomal level). DEU events were more balanced, with 10/50 genes in the overexpression (all upregulated, 6 at both levels) and 14/50 in the knock-down (4 up-, 3 down-regulated, 7 with both up- and down-regulated exons). Eventually, we intersected all datasets (Figure 3G): 38/50 genes (76%) are controlled by RBMX, 31 of which (62%) are also bound by RBMX. Of the 38 core homeotic genes, 28 are modulated (73%), 22 of which (58%) are also bound by RBMX (Supplementary Tables 6, 7, overlaps *p*-value by hypergeometric test = n.s.). This data suggests that RBMX could affect the fate of homeotic mRNAs through complementary regulatory mechanisms.

RBMX is known to control its target transcripts splicing and abundance (Liu et al., 2017), as we observed. We also detected some events at the polysomal level only (1 DEG and 4 DEUs), suggesting it may also modulate translation. We thus performed two replicates of a preliminary experiment to check RBMX polysomal localization by a sucrose gradient separation followed by a fraction-by-fraction western blot. We observed cytoplasmic RBMX mostly in polysomal fractions (Supplementary Figure S4A). There appears no enrichment in heavy or lighter polysomes, a profile typical of polysome-associated factors. However, this experiment will need further work to allow conclusively clarifying this aspect.

We eventually explored protein-protein interactions of RBMX with translation factors, reasoning that these may suggest its involvement in translation. We collected 83 experimentally determined interactions from STRING (Szklarczyk et al., 2017) and IntAct (Orchard et al., 2014) and found RBMX interactors enriched in positive regulators of translation (Supplementary Figure S4B, adjusted Fisher test *p* = 9.7E-04, Supplementary Tables 8, 9). Of those, RBM3, FXR2, and KHDRBS1 associate with polysomes (Siomi et al., 1996; Dresios et al., 2005; Paronetto et al., 2006), while CIRBP interacts with EIF4G1 (Yang et al., 2006). Post-transcriptional processes such as mRNA splicing, processing, and stabilization (23/23/4 genes, *p* = 1.21E-21, 2.6E-21, 6.3E-04) are also enriched. These results suggest a potential role in translational control for RBMX, warranting further experiments to confirm this possibility.

## Discussion

Here we used a computational approach to extract phylogenetically hyperconserved elements from the 5′UTR of human messenger RNAs (5′HCEs). We thus expanded the known catalog of regulatory mechanisms mediating the role of 5′UTRs in shaping cell phenotypes. We focused on extracting the most conserved regions, under the assumption that these would be evolutionary stable PTR sites of utmost importance.

The 5248 5′HCEs we identified are short regions occurring in 10% of protein-coding genes, mostly localized close to one another. Given their clustered nature, 5′HCEs could represent loci of cooperation and competition between post-transcriptional regulatory factors. Through their interplay, these factors would ultimately determine the translation of the containing mRNAs. As 5′HCEs do not systematically overlap with 5′UTR-specific cis-elements, binding sites for RNA-binding protein (RBP) genes are the likely orchestrators of such behaviors. Understanding how these mechanisms work and impact cell physiology and pathology will thus require further efforts toward the systematic mapping of RBP binding sites.

Among genes whose mRNA contains a 5′HCE, we identified many homeotic genes, including members of all HOX clusters and several other families. Homeotic genes are the prototypical class of conserved genes in metazoa, responsible for the development of the body plan, organs, and the nervous system (Holland, 2013). They represent the ideal result of our algorithm, benchmarking its ability to identify truly conserved regions. Identifying homeotic 5′HCEs allowed us to improve our understanding of how PTR controls development through 5′UTRs.

By an integrated *de novo* motif search and RBP motifs matching, we identified RBMX as a candidate post-transcriptional regulator of homeotic genes. We confirmed this hypothesis by probing multiple aspects of post-transcriptional regulation (PTR), including alternative splicing, mRNA stabilization, and translation. Such data hinted to the yet unappreciated involvement of RBMX in translation, to be confirmed by further studies. This would establish RBMX as a versatile controller of the mRNA, able to impact its lifecycle from alternative splicing to protein production. This multifunctional RBP can indeed be located both in the nucleus and in the cytoplasm (Matsunaga et al., 2012). Our work further corroborates the role of RBMX in development, first observed in zebrafish and frog (Tsend-Ayush et al., 2005; Dichmann et al., 2008), through the modulation of important regulators of this process. Aside from RBMX, also other RBPs may contribute to the PTR of homeotic genes. Further studies will thus be needed to complete this essential regulatory network.

## Data Availability Statement

The datasets generated for this study can be found in the Gene Expression Omnibus with IDs GSE68990 and GSE118383.

## Author Contributions

ED designed the research and performed the computational work. PZ, DP, and VP performed the experimental work. ED wrote the manuscript with inputs from AQ. All authors read and approved the manuscript.

## Conflict of Interest

The authors declare that the research was conducted in the absence of any commercial or financial relationships that could be construed as a potential conflict of interest.

## Supplementary Material

FIGURE S1 5′HCEs properties at different HCS thresholds. The figure displays abundance, length, and occupied fraction of 5′UTR space by 5′HCEs if varying the HCS threshold. **(A)** shows the number of 5′HCEs, **(B)** displays the mean length of 5′HCEs, **(C)** shows the fraction of 5′UTR space occupied by 5′HCEs.

FIGURE S2 Individual consensuses composing the motif identified in 5′HCE. The figure shows individual motifs identified in 5′HCE sequences which were identified and clustered by DynaMIT into the final motif displayed in Figure 2A. **(A)** displays the individual sequence motifs found by Weeder, aligned to highlight their similarity. Stars at the bottom indicate the columns of the alignment which are in full agreement between all sequences. **(B)** shows the secondary structure motif found by RNAforester, with sequence consensus strength shown on top and structure consensus strength at the bottom. The height of star bars corresponds to the percentage of sequence having that specific nucleotide/structure feature in that position of the motif, with “.” Indicating an unpaired nucleotide. **(C)** matches for the RBMX binding motif in the HCE portions of homeotic genes 5′UTRs. Matches, represented by orange boxes, are clustered in 15 nucleotide windows (i.e., a single orange box may include multiple matches within 15 nucleotides) for visualization purposes.

FIGURE S3 Images of full blots. **(A)** RBMX silencing blot. **(B)** RBMX overexpression blot. **(C,D)** images of fraction-by-fraction polysomes localization blots (two replicates) for RBMX and RPL26.

FIGURE S4 RBMX is potentially involved in translation. **(A)** shows the distribution of RBMX on polysomes through a western blot of the fractions derived by polysomal profiling, with the RPL26 ribosomal protein used as the positive control. Different parts of the gel are shown for the RBMX and control bands. Full blots are shown in Supplementary Figures 3C,D. **(B)** displays post-transcriptional regulatory processes enriched in RBMX protein-protein interactors. The enrichment *p*-value is shown on the *x*-axis as −log10(*p*-value). The number of RBMX interactors annotated to each process is shown next to the corresponding bar.

## Abbreviations

DEG: differentially expressed gene
DEU: differential exon usage
HCE: Hyperconserved element
HCS: Hyperconservation score
PTR: post-transcriptional regulation of gene expression
RIP: RNA immunoprecipitation.
