# Supplementary figures and images for: Hyperconserved Elements in Human 5′UTRs Shape Essential Post-transcriptional Regulatory Networks
Source: Front Mol Biosci. 2020 Aug 28;7:220. doi: 10.3389/fmolb.2020.00220 (PMC7484617; doi:10.3389/fmolb.2020.00220)

A

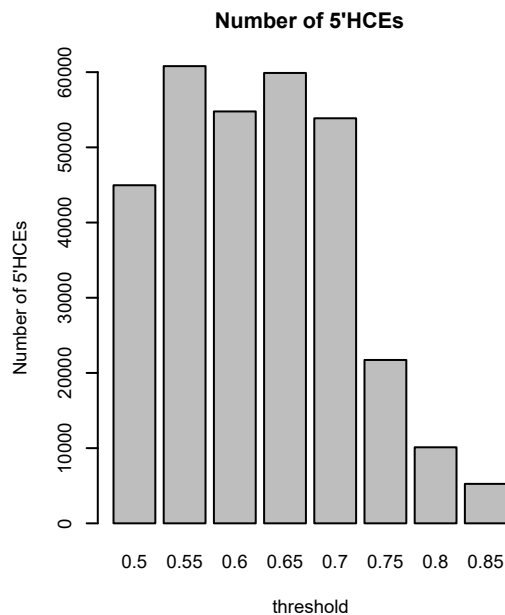

B

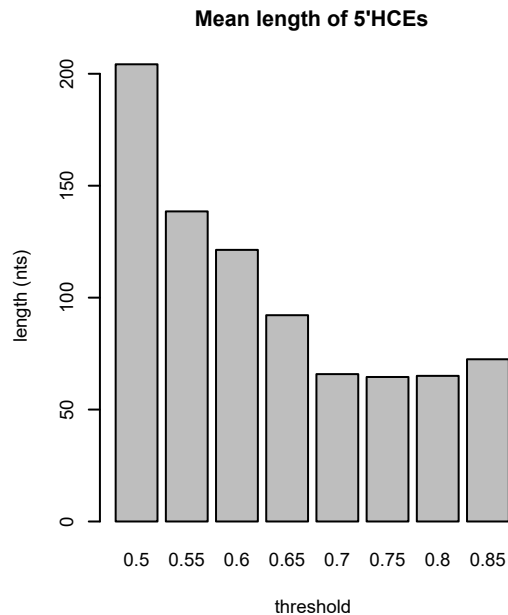

C

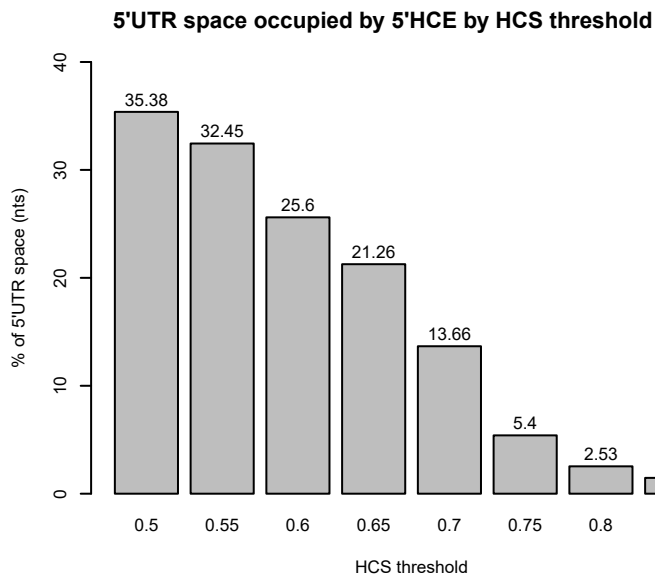

Supplement: FIGURE S1 — 5′HCEs properties at different HCS thresholds. The figure displays abundance, length, and occupied fraction of 5′UTR space by 5′HCEs if varying the HCS threshold. (A) shows the number of 5′HCEs, (B) displays the mean length of 5′HCEs, (C) shows the fraction of 5′UTR space occupied by 5′HCEs. [file Image_1.PDF]

A

RBMX

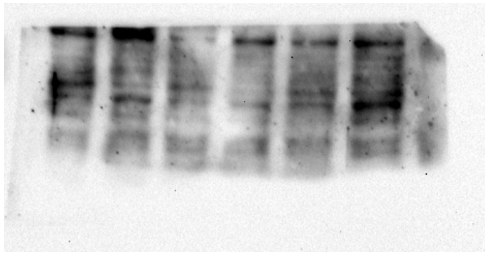

ACTININ

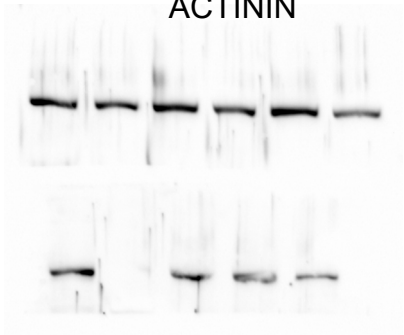

B

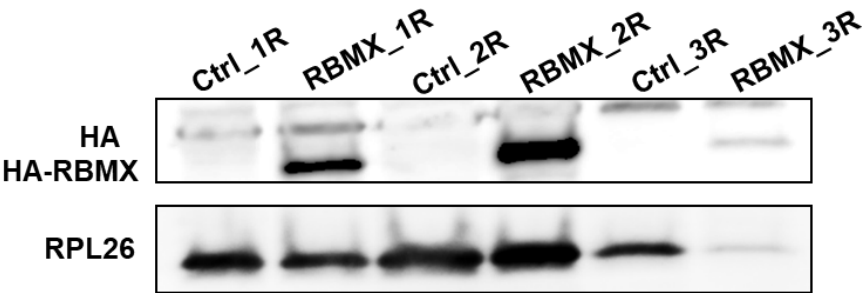

C

HA-RBMX

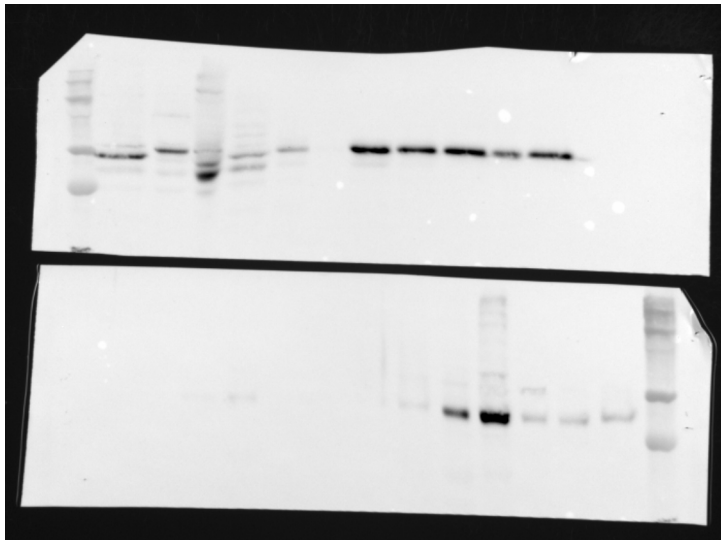

RPL26

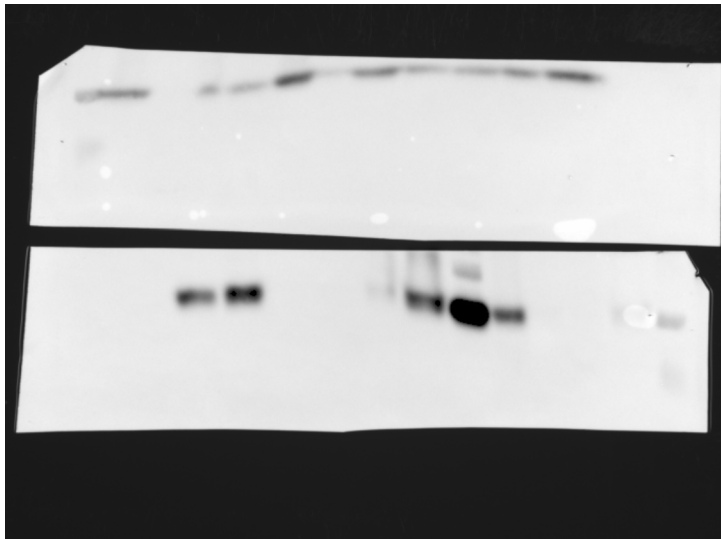

D

HA-RBMX

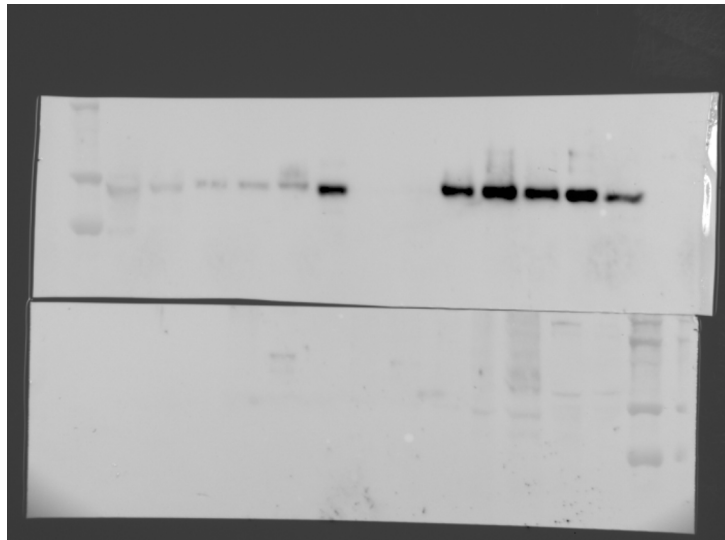

RPL26

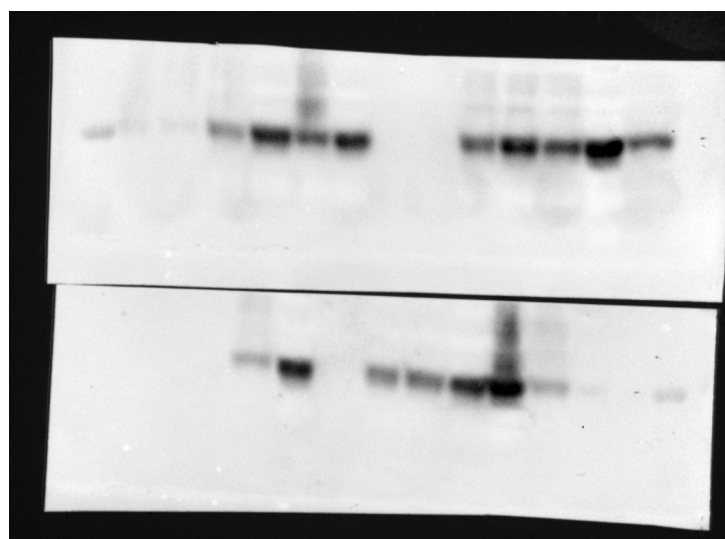

Supplement: FIGURE S3 — Images of full blots. (A) RBMX silencing blot. (B) RBMX overexpression blot. (C,D) images of fraction-by-fraction polysomes localization blots (two replicates) for RBMX and RPL26. [file Image_3.PDF]
