# Supplementary material for: Hyperconserved Elements in Human 5′UTRs Shape Essential Post-transcriptional Regulatory Networks
Source: Front Mol Biosci. 2020 Aug 28;7:220. doi: 10.3389/fmolb.2020.00220 (PMC7484617; doi:10.3389/fmolb.2020.00220)
Supplement: FIGURE S2 — Individual consensuses composing the motif identified in 5′HCE. The figure shows individual motifs identified in 5′HCE sequences which were identified and clustered by DynaMIT into the final motif displayed in Figure 2A. (A) displays the individual sequence motifs found by Weeder, aligned to highlight their similarity. Stars at the bottom indicate the columns of the alignment which are in full agreement between all sequences. (B) shows the secondary structure motif found by RNAforester, with sequence consensus strength shown on top and structure consensus strength at the bottom. The height of star bars corresponds to the percentage of sequence having that specific nucleotide/structure feature in that position of the motif, with “.” Indicating an unpaired nucleotide. (C) matches for the RBMX binding motif in the HCE portions of homeotic genes 5′UTRs. Matches, represented by orange boxes, are clustered in 15 nucleotide windows (i.e., a single orange box may include multiple matches within 15 nucleotides) for visualization purposes. [file Image_2.PDF]

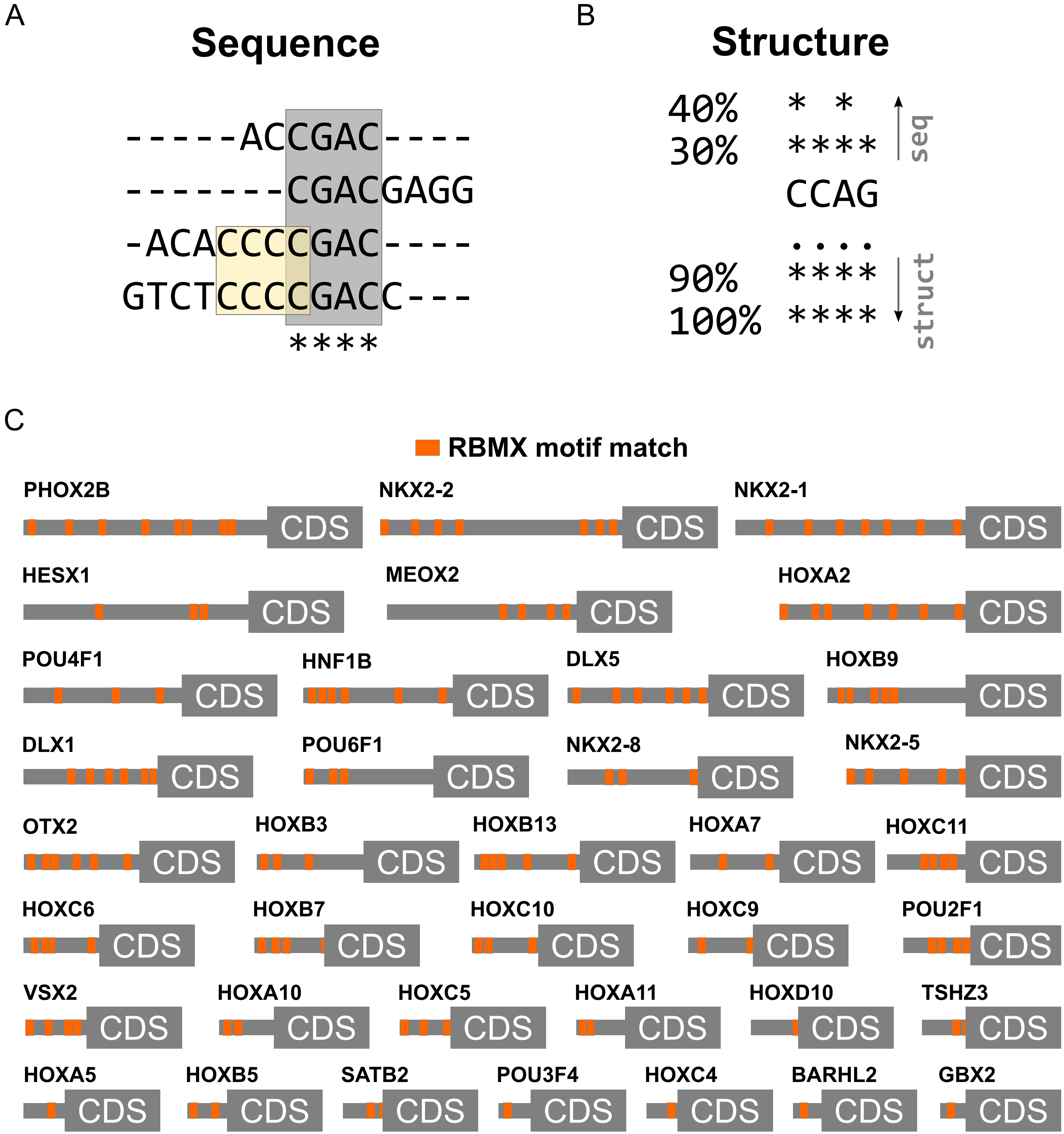

A
Sequence
-----ACCGAC----
--------CGACGAGG
-ACACCCCGAC----
GTCTCCCCGACC---
****
B
Structure
40% * *
30% ****
CCAG
....
90% ****
100% ****
seq
struct
C
RBMX motif match
PHOX2B
NKX2-2
NKX2-1
HESX1
MEOX2
HOXA2
POU4F1
HNF1B
DLX5
HOXB9
DLX1
POU6F1
NKX2-8
NKX2-5
OTX2
HOXB3
HOXB13
HOXA7
HOXC11
HOXC6
HOXB7
HOXC10
HOXC9
POU2F1
VSX2
HOXA10
HOXC5
HOXA11
HOXD10
TSHZ3
HOXA5
HOXB5
SATB2
POU3F4
HOXC4
BARHL2
GBX2
CDS
