# Supplementary material for: Hyperconserved Elements in Human 5′UTRs Shape Essential Post-transcriptional Regulatory Networks
Source: Front Mol Biosci. 2020 Aug 28;7:220. doi: 10.3389/fmolb.2020.00220 (PMC7484617; doi:10.3389/fmolb.2020.00220)
Supplement: FIGURE S4 — RBMX is potentially involved in translation. (A) shows the distribution of RBMX on polysomes through a western blot of the fractions derived by polysomal profiling, with the RPL26 ribosomal protein used as the positive control. Different parts of the gel are shown for the RBMX and control bands. Full blots are shown in Supplementary Figures 3C,D. (B) displays post-transcriptional regulatory processes enriched in RBMX protein-protein interactors. The enrichment p-value is shown on the x-axis as −log10(p-value). The number of RBMX interactors annotated to each process is shown next to the corresponding bar. [file Image_4.PDF]

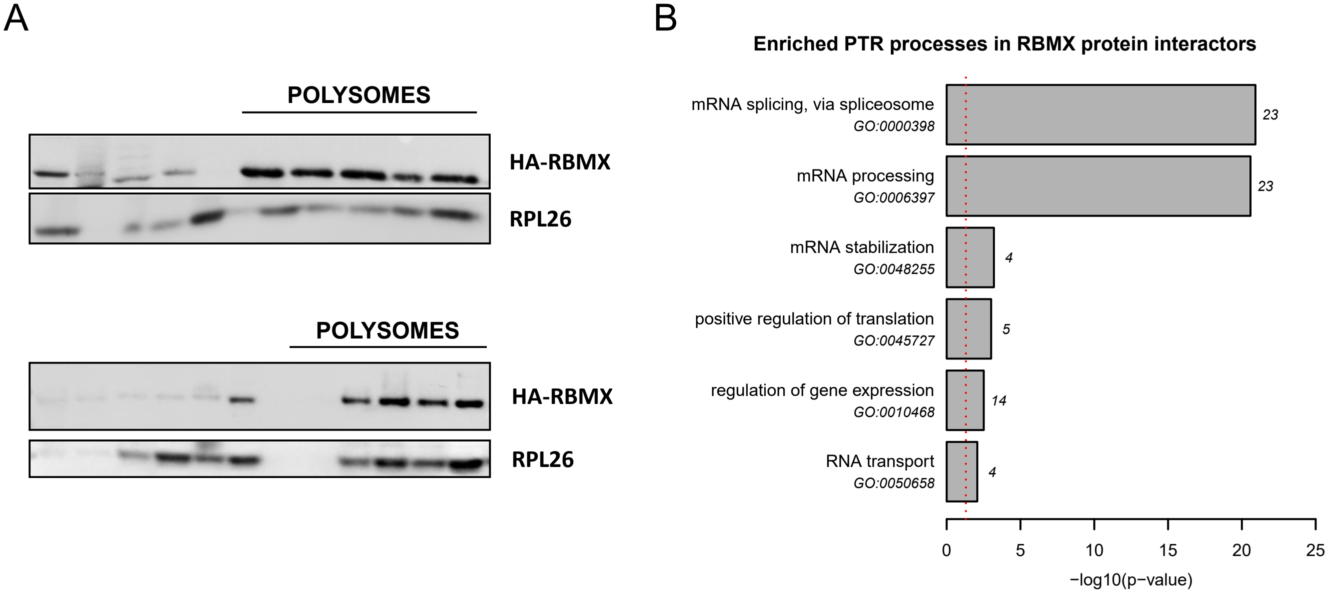
A
POLYSOMES
HA-RBMX
RPL26
POLYSOMES
HA-RBMX
RPL26
B
Enriched PTR processes in RBMX protein interactors
mRNA splicing, via spliceosome
GO:0000398
23
mRNA processing
GO:0006397
23
mRNA stabilization
GO:0048255
4
positive regulation of translation
GO:0045727
5
regulation of gene expression
GO:0010468
14
RNA transport
GO:0050658
4
0
5
10
15
20
25
−log10(p−value)
